# Social cognition in young adults who endorse a cannabis use disorder

**DOI:** 10.1007/s00213-025-06890-z

**Published:** 2025-09-17

**Authors:** Gabrielle Abbott, Lisa-Marie Greenwood, Jessica G. Bartschi, Suraya Dunsford, Isabella Goodwin, Anastasia Paloubis, Marianna Quinones-Valera, Eugene McTavish, Antonio Verdejo-Garcia, Janna Cousijn, Gary C. K. Chan, Nadia Solowij, Valentina Lorenzetti

**Affiliations:** 1 School of Psychology, Faculty of the Arts, Social Sciences, and Humanities, University of Wollongong, Wollongong, Australia; 2 School of Medicine and Psychology, The Australian National University, Canberra, Australia; 3 School of Psychology, Faculty of Health, University of Plymouth, Plymouth, UK; 4 Brain Research and Imaging Centre, Faculty of Health, University of Plymouth, Plymouth, UK; 5 Neuroscience of Addiction and Mental Health Program, Healthy Brain and Mind Research Centre, School of Health and Behavioural Sciences, Faculty of Health Sciences, Australian Catholic University, Level 5 Daniel Mannix Building, 115 Victoria Parade, Fitzroy, VIC 3065 Australia; 6 School of Psychological Sciences and Turner Institute for Brain and Mental Health, Monash University, Melbourne, Australia; 7 Neuroscience of Addiction Lab, Department of Psychology, Education & Child Studies, Erasmus University Rotterdam, Rotterdam, The Netherlands; 8 National Centre for Youth Substance Use Research, University of Queensland, Brisbane, Australia; 9 Clinical Psychopharmacology Unit, Research Department of Clinical, Educational and Health Psychology, University College London, London, UK

**Keywords:** Cannabis, Cannabis use disorder, Social cognition, Dosage, Emotion

## Abstract

**Rationale:**

Cannabis use disorder (CUD) affects over 50 million people globally. Emerging evidence shows that some people with CUD may experience altered social cognition (e.g., emotion recognition or differentiation). These impairments can affect their ability to understand others’ emotional states and navigate social interactions, potentially contributing to chronic cannabis use, even when it leads to interpersonal problems. However, the literature on social cognition in cannabis users is inconsistent, based on a paucity of studies, and characterised by methodological issues including conflation of remitted and current CUD (i.e., does not consider abstinence effects on cognition), limited assessment of cannabis metrics (e.g., dosage) and confounds entrenched with CUD (e.g., nicotine/alcohol use, anxiety).

**Objectives/methods:**

We aimed to examine social cognition (i.e., emotion recognition and differentiation, immediate/delayed face memory) in relation to endorsement of CUD (*n* = 83) vs. controls (*n* = 32), and measures of level of problematic cannabis use (i.e., Cannabis Use Disorder Identification Test – Revised; CUDIT-R) and dosage (i.e., cannabis grams/past month), accounting for hours since last cannabis use, nicotine/alcohol use, and trait anxiety.

**Results:**

There were no significant effects of CUD (*d* = 0–0.314) or dosage and level of problematic cannabis use on social cognition.

**Conclusions:**

Altered social cognition may not be a key feature of CUD, or the relationship between CUD and cognition may be moderated by factors such as age, treatment seeking, education, and IQ. In this study, younger age and higher education or IQ may have served as protective factors against social alterations. Replication studies are required to validate this notion.

**Supplementary Information:**

The online version contains supplementary material available at 10.1007/s00213-025-06890-z.

## Introduction

Around 50 million individuals experience a cannabis use disorder (CUD) worldwide (Leung et al. 2020). The global prevalence of CUD has increased by 32% from 1990 to 2019 (Shao et al. 2023), and currently CUDs account for 49% of all substance use disorders worldwide (UNODC 2024). These statistics are concerning, as CUD can be characterised by recurrent use despite difficulties with interpersonal, occupational, and/or social functioning as a consequence of cannabis consumption (DSM-5-TR 2022). In line with cognitive theories of addiction, the psychosocial difficulties entrenched with CUD have been partly ascribed to altered social cognition (Koob 2015; Koob and Volkow 2016). Emerging evidence suggests a bidirectional relationship between altered social cognition and CUD, whereby impaired social cognition, such as diminished emotion recognition or differentiation, may contribute to persistent use which then further exacerbates these deficits, thus reinforcing the cycle of addiction (Goldstein and Volkow 2011; Hammond et al. 2022).

Social cognition encompasses a range of functions, from recognising faces to identifying and differentiating emotions (Arioli et al. 2018). Social cognitive functions underpin the ability to understand others’ internal states (e.g., thoughts, emotions) and build/maintain interpersonal relationships (Arioli et al. 2018; Hess 2016). Notably, impairments in social cognition (e.g., emotion recognition/differentiation, face memory) have been implicated in the development and maintenance of substance use disorders, including CUD (Koob 2015; Koob and Volkow 2016) and mental health problems associated with CUD (e.g., anxiety, depression) (Baez et al. 2023; Demenescu et al. 2010; Onaemo et al. 2021).

Early evidence shows altered social cognition in cannabis users (MacKenzie et al. 2023). For example, cannabis users compared to controls, took more time to identify sadness, happiness, and anger (i.e., Dynamic Emotional Expression Recognition Task; DEER-T) (Platt et al. 2010) and were less accurate as measured by hit rate and sensitivity (i.e. Emotion Processing Task) (Hindocha et al. 2014). Similarly, individuals with CUD compared to controls have shown worse identification of negative emotions (e.g., sadness, shame) and worse discrimination of positive and negative emotions (Bayrakçı et al. 2014) during the Facial Emotion Discrimination Test (FEDT) and Facial Emotion Identification Test (FEIT) (Bayrakçı et al. 2014). Whereas another study found no group differences between CUD and controls in domains of social cognition including face memory and emotion recognition (Koenis et al. 2021).

In addition, early evidence shows that level of problematic cannabis use may be associated with worse social cognition; with greater scores on the Cannabis Use Disorder Identification Test (CUDIT) being associated with poorer emotion recognition of sadness, fear, and anger (Blair et al. 2021). Yet, other research in cannabis users found no significant correlations between frequency, dosage, duration of cannabis use, and emotion identification or emotion differentiation (Bayrakçı et al. 2014). Minimal other research has examined the effect of dosage on social cognition which may be influenced by the lack of consensus for measuring quantities of use (e.g., joints per week, hits past year) (Bayrakçı et al. 2014; Becker et al. 2018).

There are other methodological limitations in the available evidence on social cognition in CUD. First, only a few studies to date have examined social cognition in regular cannabis users with CUD (Bayrakçı et al. 2014; Blair et al. 2021; Koenis et al. 2021). Thus, the evidence base is inadequate to determine whether social cognition is a key feature of CUD or a byproduct of cannabis intoxication and subacute effects that should be accounted for in preventative interventions and treatment settings (Kroon et al. 2020). Second, few studies have examined participants from the general community who endorse current CUD and who are currently using cannabis, which comprise the majority of people who use cannabis, as less than 15% are estimated to seek or receive treatment (Kerridge et al. 2017). Indeed, CUD samples in previous studies were treatment seekers and often abstinent (Bayrakçı et al. 2014; Blair et al. 2021; Koenis et al. 2021) which calls into question the generalisability of these findings to current users.

Third, few studies have examined other metrics of cannabis use in CUD such as whether level of cannabis related problems or dosage affects social cognition in those who endorse a CUD (Bayrakçı et al. 2014; Blair et al. 2021). Given previous early evidence that greater severity of problematic cannabis use (i.e., CUDIT) affects emotion recognition (Blair et al. 2021), it is important to understand if this notion is replicable and extends to other aspects of social cognition (i.e., emotion differentiation) to identify who are the most vulnerable consumers. Finally, confounding variables entrenched with cannabis use that impact cognition independently and in interaction with cannabis use have not been consistently accounted for (e.g., alcohol and nicotine use, anxiety symptoms, hours since last cannabis use) (Copersino 2017; Hofmann et al. 2012; Koob 2015; Koob and Volkow 2016; Verdejo-Garcia et al. 2019); and it is unclear if alterations are due to CUD or confounders entrenched with use. Importantly social cognitive deficits may negatively impact interpersonal relationships and increase the likelihood of social isolation (Tracy and Wallace 2016). Therefore, new evidence in CUD is required to clarify the association between CUD and social cognition, to better understand how to support individuals who experience a CUD.

The primary aim of the current study was to determine whether distinct aspects of social cognition (i.e., emotion identification, emotion differentiation, immediate and delayed face memory) differ between regular cannabis users who endorse a CUD and controls, accounting for alcohol and nicotine use, and trait anxiety. The secondary aim of the study was to explore whether level of problematic cannabis use and dosage (i.e., CUDIT-R scores, cannabis grams/past month) is associated with social cognition (i.e., emotion identification, emotion differentiation, immediate and delayed face memory) in the CUD group, accounting for alcohol and nicotine use, trait anxiety, and hours since last cannabis use. Based on emerging evidence, it was hypothesised that the presence of a CUD, CUD-related problems and cannabis dosage, would be associated with worse performance in emotion differentiation and recognition. We explored the association between CUD, CUD-related problems and cannabis dosage and immediate/delayed face memory.

## Methods

### Recruitment

This study occurred within a larger project. Participants (*N* = 136) were recruited via advertisements posted around University of Wollongong campuses and online social media platforms. Informed consent was obtained from all participants and ethics was approved by the University of Wollongong Human Research Ethics Committee (HREC: 2017/389). Participants were reimbursed $40 AUD for completing the face-to-face testing session.

### Participant inclusion and exclusion criteria

#### Inclusion criteria

Participants were required to be aged 18–55, fluent in English, and have an intelligence quotient (IQ) ≥ 80. Cannabis users were further required to (i) endorse Diagnostic and Statistical Manual for Mental Disorders – Fifth Edition (DSM-5-TR 2022) criteria for a CUD (i.e., scoring ≥ 2 on the Structured Clinical Interview for DSM-5 – Research Version; SCID-5-RV) (First et al. 2015), and (ii) currently use cannabis ≥ 4 days per week, and reported using at this level on average for 12-months prior to testing.

#### Exclusion criteria

Participants were excluded for the following reasons: (i) self-reported or met DSM-5 criteria on the Mini International Neuropsychiatric Interview (MINI-5) (Sheehan et al. 1998) for current or past diagnosis of psychotic illness, bipolar disorder I and II, obsessive-compulsive disorder, current severe alcohol or other substance use disorder, or self-reported past treatment for alcohol use or drugs other than cannabis; (ii) self-reported high nicotine dependence on the Fagerström Test for Nicotine Dependence (FTND, ≥ 8) (Fagerström 2012), or high risk for alcohol related harm on the Alcohol Use Disorder Identification Test (AUDIT, ≥ 20) (Babor et al. 2001); (iii) currently pregnant or breastfeeding; (iv) lifetime use of illicit substances exceeding recreational levels (other than cannabis for CUD group), measured by the Drug History Questionnaire (DHQ) (Sobell et al. 1995), defined as: weekly use for ≥ 3 months within the past 5 years; cumulative lifetime episodes > 50; and cumulative lifetime use > 5 for methamphetamine or any drug administered intravenously; (v) use of any illicit substance (other than cannabis for CUD group) within 30-days prior to testing; and (vi) known contraindications for cognition data interpretation (e.g., history of neurological illness such as encephalitis or moderate to severe brain injury). Controls were excluded if they reported (i) cannabis use within 3-months prior to testing; (ii) ≥ 50 lifetime cannabis use occasions, or (iii) a history of weekly or more frequent cannabis use over a ≥ 3-month period.

Participants abstained from using cannabis and alcohol 12-hours prior to testing to elucidate non-intoxicated residual effects of cannabis on cognition. A urine sample was collected for toxicology assessment (ECO II – Multi-Drug Screen: amphetamine, benzodiazepine, cocaine, cannabis, methamphetamine, opiate) to ensure the presence of delta-9-tetrahydrocannabinol (Δ9-THC) metabolites in the CUD sample, to detect other illicit substance use, and for pregnancy testing (Alere hCG Cassette). A breath analyser (Alcolizer Easy Check - HH1) was used to corroborate self-reported alcohol abstinence (BAC 0.00%). Any discrepancies between test results and reports of recent drug or alcohol use were clarified with participants. All inclusion and exclusion criteria were assessed prior to testing (described below) and confirmed at face-to-face testing.

Of the 136 participants recruited; seven endorsed cannabis use levels (i.e., frequency and duration of use) below the threshold for inclusion, and 14 did not complete the full face-to-face testing session due to identification of exclusionary issues or did not complete cognitive testing due to technical difficulties. This resulted in a final sample of 115 participants included for analysis. Twenty-nine participants reported past use of illicit substances (other than cannabis for the CUD sample) above defined recreational levels and/or within 30-days prior to testing (Supplementary Table 1). Visual and statistical inspection of distributions of social cognition outcome variables suggested these participants were *not* outliers or contributing to skewed data. As a result, these participants were retained in analyses to represent members of the community who consume cannabis on a regular basis and other illicit substances occasionally (Hasin and Walsh 2020).

### Procedure

Interested members of the community completed an online questionnaire and telephone screening to assess eligibility prior to the face-to-face testing session (see Supplementary Material section 1.1and 1.2). Eligible participants completed a 3-hour testing session at the research facilities located in the School of Psychology at the University of Wollongong. Participants completed semi-structured interviews that asked questions about current and past cannabis use, lifetime history of other substance use using the DHQ (Sobell et al. 1995), recent drug use using the Timeline Follow-Back (TLFB) (Robinson et al. 2014; Sobell and Sobell 1992), and general demographic questions. The absence of exclusionary current and past psychopathology was reconfirmed using the MINI-5 (Sheehan et al. 1998). A computer-based self-report questionnaire battery was administered to reassess levels of nicotine dependence (FTND; scores ≥ 8) (Fagerström 2012) and problematic alcohol use (AUDIT; scores ≥ 20) (Babor et al. 2001) and ensure they did not violate exclusion criteria, and to assess additional sample characteristics including schizotypal traits (i.e., Schizotypal Personality Questionnaire; SPQ) (Raine 1991) and psychotic experiences (i.e., Community Assessment of Psychic Experiences; CAPE) (Stefanis et al. 2002). The State-Trait Anxiety Inventory (STAI-Trait) (Spielberger 1989; Spielberger et al. 1983) assessed general trait anxiety and was used as a covariate in all analyses.

All cannabis users were re-administered the ‘alcohol and other substance use disorders’ module of the SCID-5-RV (First et al. 2015) during the semi-structured interview to determine CUD symptoms. A semi-structured interview was used to ascertain cannabis use history and characterise current usage levels. From this information we extracted participants’ age at onset of regular use (i.e., age when consuming cannabis at least 4 days per week for 3-months or more). The TLFB was used to assess recent cannabis use metrics, specifically to determine total cannabis grams and use days in the month prior to testing, as well as hours since last cannabis use. The Cannabis Use Disorder Identification Test – Revised (CUDIT-R) (Adamson et al. 2010; Adamson and Sellman 2003) assessed problematic cannabis use patterns and was used as a predictor variable for analyses in the CUD sample. Full-scale IQ was measured via the Wechsler Abbreviated Scale of Intelligence – Second Edition (WASI-II), including the matrix reasoning and vocabulary subtests (Wechsler 2011) which was analysed as a demographic variable. Following completion of interviews and self-report measures, all participants underwent social cognitive testing.

#### Social cognitive testing

Social cognition was assessed for a range of domains (i.e., emotion recognition, emotion differentiation, immediate and delayed face memory) using the adult Pennsylvania Computerised Neurocognitive Battery (Penn CNB) (Gur et al. 2001, 2010). The adult Penn CNB includes a number of subtests that assess various domains of cognition; four subtests capturing aspects of social cognition (Moore et al. 2015) were administered as part of the current study. The four subtests were: (i) emotion recognition (ER): a measure of emotion identification where participants were shown a series of 40 faces and were asked to determine what emotion the face was showing by selecting one of the five emotion labels (i.e., happy, sad, anger, fear, no emotion) (ii) measured emotion differentiation (MED): a measure of emotion differentiation where participants were shown 40 pairs of faces and asked to determine whether one of the faces expressed the named emotion (i.e., happy or sad) more intensely or whether they were equal in emotional intensity, and (iii/iv) immediate face memory (FM) and delayed (FM-d): a measure of facial episodic memory where participants were shown 20 faces and were then asked to identify those faces in a series of 40 faces (i.e., 20 target stimuli and 20 novel) at an immediate and delayed (15–30 min later) recall interval.

The Penn CNB was administered in a standardised lab environment with a consistent set-up for desk height, and mouse, keyboard and monitor size (i.e., 62 centimetres, measured diagonally from bottom left corner to top right corner). For all Penn CNB subtests, accuracy (i.e., total number or percent correct) and speed scores (i.e., median response time, milliseconds) were transformed to their standardised equivalent (i.e., z-score) and combined to calculate an efficiency score, consistent with the literature (Merikangas et al. 2017; Moore et al. 2015; Van Pelt et al. 2021). All z-scores for median response time were multiplied by −1 to produce a speed value where, as for accuracy, higher scores reflect better performance.

### Statistical analyses

All analyses were performed using IBM SPSS Statistics (Version 29) (IBM Corp., 2022), R (Version 4.4.0) (R Core Team, 2024), and Jamovi (Version 2.6.0) (The Jamovi Project, 2024). The level of significance was set at *p* <.05. Normality checks of descriptive and outcome data, data handling, and assumption testing is outlined in Supplementary Materials section 1.4.

#### Primary aim: social cognition differences between CUD and control group

We ran a series of one-way analyses of covariance (ANCOVA) using group (CUD vs. control) as a predictor, social cognition efficiency scores as outcome variables that were normally distributed, including emotion recognition (i.e., ER); emotion differentiation (i.e., MED); and immediate face memory (i.e., FM). A robust regression was used to examine group differences for delayed face memory (i.e., FM-d) which had a skewed distribution. All analyses were run with and without adjusting for covariates including nicotine use (i.e., FTND), alcohol use (i.e., AUDIT), and trait anxiety (i.e., STAI-Trait).

#### Secondary aim: CUDIT-R and cannabis grams/past month predicting social cognition in the CUD group

In the CUD group, regression analyses were used to determine whether the predictor variables, level of problematic cannabis use (i.e., CUDIT-R scores) and cannabis dosage (i.e., cannabis grams/past month), predicted outcome variables that were normally distributed including: emotion recognition (i.e., ER); emotion differentiation (i.e., MED); immediate face memory (i.e., FM). A robust regression was run to examine the effect of CUDIT-R and cannabis grams/past month as predictors on delayed face memory (i.e., FM-d) due to its skewed distribution. All analyses were run with and without adjusting for covariates including nicotine use (i.e., FTND), alcohol use (i.e., AUDIT), trait anxiety scores (i.e., STAI-Trait), as well as number of hours since last cannabis use.

#### Sensitivity analyses

To confirm the robustness of results, sensitivity analyses were conducted to examine the primary and secondary aims, excluding outliers defined as +/- 3 standard deviations from the mean (see Supplementary Table 2).

## Results

### Sample descriptives

Sample characteristics are shown in Table 1. This study included a sample of 115 participants (82 male, 33 female) with a mean age of 23.2 years. Of these, 83 participants endorsed a CUD and 32 were controls. The CUD group compared to controls had significantly fewer years of education, lower IQ, and showed significantly higher trait anxiety, schizotypal personality traits, and psychotic experiences. The CUD group also had significantly greater FTND and AUDIT scores, as well as reported consuming a higher number of standard alcoholic drinks and cigarettes in the previous month.

**Table 1:** Mean (standard deviation) and median [range] for demographic, substance use, and psychopathology characteristics by group

	CUD M (SD)	Median [range]	Control M (SD)	Median [range]	Group differences t/c^2^,^df^ *p*
Sex, N [female]	83 [21]	-	32 [12]	-	20.9^1^, **< 0.001**, -
Age, years	23.6 (3.36)	22.5 [14]	22.3 (2.81)	21.5 [10.1]	3.59^1^, 0.058, -
Education, years	15 (1.88)	15 [9]	15.9 (1.92)	15.8 [8)	4.72^1^, **0.030**, -
IQ	116 (10.7)	117 [49]	125 (9.22)	127 [38]	14.5^1^, **< 0.001**, -
Trait anxiety (STAI)	38.3 (9.88)	36.5 [41]	32 (8.61)	31 [38]	−3.06^1^, **0.003**, -
Schizotypy (SPQ)	22.2 (10.2)	21 [47]	10.7 (7.52)	9.5 [25]	27.2^1^, **< 0.001**
CAPE Total	90 (22.9)	88 [90]	66.4 (20.8)	62 [96]	25.2^1^, **< 0.001**
CAPE positive freq	24.9 (3.52)	24 [13]	21.4 (1.54)	21 [5]	28.4^1^, **< 0.001**
CAPE negative freq	23.4 (5.17)	23 [21]	19.4 (5.11)	18.5 [26]	16.4^1^, **< 0.001**
CAPE depressive freq	13.5 (3.23)	13 [12]	11.3 (2.86)	11 [12]	12.7^1^, **< 0.001**
FTND	0.964 (1.34)	0 [6]	0.313 (0.821)	0 [4]	9.0^1^, **0.003**, -
N cigarettes/past month	38.2 (73.4)	2 [285]	0.35 (1.4)	0 [7]	18.81^1^, **< 0.001**
AUDIT	6.65 (4.72)	6 [22]	3.94 (3.26)	3.5 [12]	8.48^1^, **0.004**, -
N drinks/past month^a^	25.8 (31)	15 [161]	8.84 (10.9)	5 [38.5]	8.34^1^, **0.004**
*Cannabis use*					
Age of first use	16.7 (2.64)	16.2 [16.3]	-	-	-
Age of regular use,	19.9 (2.84)	19.8 [15.3]	-	-	-
Days/past month	24.5 (4.58)	26 [22]	-	-	-
Dose, grams/past month^b^	20.1 (23.6)	11.5 [129]	-	-	-
CUD symptoms, SCID-5-RV	5.35 (2.3)	5 [8]	-	-	-
CUDIT-R	17.8 (5.24)	18 [23]	-	-	-
THC-COOH in urine, ng/ml	63.96 (75.11)	32.84 [372.97]			
Abstinence, hours	26.9 (57)	14.3 [504]	-	-	-

bold = *p *< .05; a = standard alcoholic units; b = total grams over the past 30-days; age of regular cannabis use was defined by the age when participants started using 4 days per week for at least 12-months; freq = frequency; - = NA or not provided by statistical output; IQ = Intelligence Quotient; STAI = State-Trait Anxiety Inventory; SPQ = Schizotypal Personality Questionnaire; CAPE = Community Assessment of Psychic Experiences; FTND = Fagerstrom Test of Nicotine Dependence; AUDIT = Alcohol Use Disorder Identification Test; SCID-5-RV = Structured Clinical Interview for the DSM-5– Research Version; CUD = Cannabis Use Disorder; CUDIT-R = Cannabis Use Disorder Identification Test – Revised; THC-COOH = 11-nor-9-carboxy-Δ9-tetrahydrocannabinol; ng/mL = nanograms per millilitre

### Cannabis use levels

The CUD group reported First using cannabis at 16.7 years. Regular use, defined as 4 or more days per week, commenced at 19.9 years. The CUD group also reported using cannabis on an average of 24.5 out of 30 days per month, consumed an average of 20 g of cannabis in the past month, and average THC-COOH urine levels of 64 ng/ml confirm the presence of Δ9-THC metabolites. The CUD group reported that their last cannabis use occurred 14.5 h (median) prior to testing.

### Group comparison (CUD vs. controls) in social cognition

As shown in Table 2, groups did not differ significantly for social cognition outcomes, accounting for nicotine and alcohol use, and trait anxiety. Specifically, CUD and controls performed similarly for emotion recognition (*p* =.859, *d =* 0), where results remained non-significant after removing outliers (*p* =.988). Similarly, there were no significant differences between the CUD and control group for emotion differentiation (*p* =.121, *d =* 0.314, outlier removal sensitivity analysis: *p* =.194) or immediate face memory (*p* =.635, *d* = 0.090, outlier removal sensitivity analysis: *p* =.494). The robust regression examining delayed face memory found no significant differences between the CUD and control group (*p* =.273) (see Supplementary Material section 2.1.1).

**Table 2:** Social cognition differences between the CUD and control groups

		CUD M (SD)	Median [range]	Control M (SD)	Median [range]	Group comparisons t/c^2^/F^df^, *p*, d	Covariates AUDIT *p*, d	STAI-Trait *p*, d	FTND *p*, d
Emotion recognition	ER	0.019 (1.52)	0.187 [8.53]	−0.049 (1.48)	0.085 [5.86]	0.032^99^, 0.859, 0	0.771, 0.063	0.582, 0.110	**0.014**, 0.505
Emotion differentiation	MED	−0.198 (1.34)	−0.037 [7.8]	0.508 (1.27)	0.619 [4.97]	2.488^100^, 0.121, 0.314	0.701, 0.063	0.088, 0.346	0.380, 0.180
Face memory, immediate	FM	−0.037 (1.47)	0.097 [8.52]	0.095 (1.62)	0.503 [8.09]	0.226^101^, 0.635, 0.090	0.926, 0	0.674, 0.090	0.246, 0.230
delayed	FM-d	−0.065 (1.26)	0.072 [4.96]	0.168 (1.37)	0.516 [5.43]	−1.101^101^, 0.273, -	0.716, -	0.493, -	0.208, -

bold = *p *< .05; - = NA or not provided by statistical output; *d = *Cohen’s d^*^Cohen (1988) reports the following intervals: 0 - .2: very small effect; .2 to .5: small effect; .5 to .8: medium effect; .8 and higher: large effect; CUD = Cannabis Use Disorder; AUDIT = Alcohol Use Disorder Identification Test; STAI = State-Trait Anxiety Inventory; FTND = Fagerstrom Test of Nicotine Dependence

The removal of covariates resulted in a significant change to the model for emotion differentiation (*p* =.012, *d =.*487), whereby the CUD group performed significantly worse than controls (*MD* = 0.707, *SE* = 0.275). No covariates were highly correlated with emotion differentiation (ρ = 0–0.21). Results from group comparisons examining other domains of social cognition (i.e., emotion recognition, immediate and delayed face memory) remained non-significant after the removal of covariates (see Supplementary Table 3).

### CUDIT-R scores and cannabis dosage as predictors of social cognition in the CUD group

There was no significant effect of CUDIT-R scores and cannabis grams/past month on emotion recognition, emotion differentiation, or immediate and delayed face memory (*p* >.1). All results remained unaltered with and without covariates or outliers (see Supplementary Material, section 2.2.1 and Supplementary Tables 4 & 5).

## Discussion

The current study examined social cognition in non-treatment seeking regular cannabis users who endorsed a CUD and non-using controls. This study also examined social cognition in the CUD group in relation to level of problematic cannabis use (i.e., CUDIT-R scores) and cannabis dosage (i.e., cannabis grams/past month), comprehensively accounting for confounders (i.e., nicotine/alcohol use, trait anxiety, and hours from last cannabis use). In contrast to the study hypotheses, which were based on neuroscientific theories of addiction (Goldstein and Volkow 2011; Koob 2015), there was no significant effect of CUD, level of problematic cannabis use, or dosage on emotion differentiation or recognition, nor other domains of social cognition (i.e., immediate and delayed face memory), with effect sizes ranging from very small to small (i.e., *d* = 0–0.314). These results may indicate that social cognition is not a core feature of CUD or that the relationship between CUD and social cognition is influenced by moderators such as age, treatment seeking, education level, and IQ. Indeed, the current CUD sample included mostly young adults (*M* = 23.6 years) who were university students at the time of testing and had above average IQ and no participant was seeking or receiving treatment for their cannabis use. It may be that young age, access to tertiary education, high cognitive function and the lack of seeking/receiving treatment and protected participants against social cognitive alterations. Future work is required to determine if social cognition is affected in adolescents and older adults with a CUD who are seeking or undergoing treatment.

The lack of significant social cognitive differences between the CUD and control group align with a previous study that reported no group differences for some of the same measures examined herein (i.e., immediate and delayed face memory, emotion recognition) (Koenis et al. 2021). However, our results contrast with other research that found worse emotion recognition and differentiation performance in regular cannabis users (Hindocha et al. 2014; Platt et al. 2010) and former cannabis users with CUD (Bayrakçı et al. 2014) compared to controls. It is possible that inconsistent accounting for confounds known to be comorbid with cannabis use and CUD (e.g., alcohol use, nicotine use, depression and anxiety scores) across studies has contributed to mixed findings in the literature. Indeed, in the current study when covariates (i.e., alcohol and nicotine use, trait anxiety) were removed for a sensitivity analysis comparing emotion differentiation between CUD and controls, an effect emerged whereby CUD performance was significantly lower than controls. Further, two of the three studies that reported significantly worse social cognition in people who use cannabis compared to controls failed to account for potential confounding variables in analyses (i.e., depression symptoms) (Bayrakçı et al. 2014; Platt et al. 2010). The third study accounted for sex and schizotypy scores in sensitivity analyses, however, did not covary depression scores or alcohol use which may have produced an effect when entered into statistical models due to being significantly higher in the cannabis group (Hindocha et al. 2014). Our results highlight the importance of accounting for potential confounders when examining social cognition in CUD. Without our conservative approach to analyses, we may have inadvertently overstated cannabis-related effects on emotion differentiation, which may explain findings in previous studies that used less conservative statistical approaches.

Similarly, in the CUD group there was no significant relationship between level of problematic cannabis use (i.e., CUDIT-R scores) or dosage (i.e., cannabis grams/past month) and social cognition. The role of severity of use and cannabis use levels is an understudied area of the literature, which limits the interpretation of findings. The limited previous research found no association between dosage (i.e., joints/week) and emotion identification and discrimination (Bayrakçı et al. 2014), which is consistent with the current findings. However, our findings contrast with another study that found significant associations between CUDIT scores and emotion recognition (i.e., sadness, fear, and anger) in a sample of abstinent individuals with CUD (Blair et al. 2021). It may be that different characteristics between the current CUD sample and those in previous studies, such as treatment seeking, comorbidities (e.g., psychopathology, other substance use) education, and IQ, explain the inconsistent findings. First, the two previous studies that examined associations between cannabis use metrics and social cognition, utilised treatment seeking samples (Bayrakçı et al. 2014; Blair et al. 2021) that endorsed psychiatric and other substance use comorbidities (Blair et al. 2021). It may be that comorbidities may moderate social cognitive alterations however, this was not examined in the current study due to our stringent exclusion criteria, implemented to ensure sound methodology to detect cannabis-specific effects, resulting in a non-treatment seeking sample with minimal comorbidities.

Second, the current study assessed CUD symptoms and level of problematic cannabis use using robust methodology - including but not limited to SCID-5-RV for confirming the presence of a CUD, CUDIT-R to measure CUD-related problems, and timeline follow-back to measure recent exposure to cannabis. The CUD sample examined were not seeking or receiving treatment and therefore future work is required to elucidate if treatment status moderates the association between CUD and social cognition performance. Finally, our CUD sample had high levels of education and an average IQ (*M* = 116, *SD* = 10.7) one standard deviation above the mean which may indicate sampling bias. Although our study had a scope of regular cannabis users aged 18–55 years, our recruitment advertising was mostly concentrated to university campuses and associated online social media pages. It is likely that this contributed to a sample of more highly educated (*M* = 15 years, *SD* = 1.88 years), young (*M* age = 23.6 years), university students whose cannabis use may not be as severely impacting functioning compared to those seeking treatment. Indeed, CUD samples in previous research had substantially lower years of education (e.g., *M* = 8.3 years, *SD* = 2.6 years) (Bayrakçı et al. 2014) and IQ (*M* = 101.69, *SD* = 11.65) (Blair et al. 2021) compared to the current CUD sample. It may be that cannabis use has minimal effect on social cognition in people with higher IQ and related socioeconomic characteristics; however, this needs to be determined via more research.

### Limitations

First, our CUD sample characteristics may indicate the presence of sampling bias. Indeed, the CUD group had above average IQ, were young, and had minimal psychopathology and other substance use comorbidities. It may be that these findings do not reflect characteristics of social cognitive functioning of the wider CUD population with older ages or youth, treatment seekers and polysubstance users. To ensure research is generalisable to CUD populations, future research should recruit participants with a range of educational experiences and psychosocial characteristics (e.g., comorbid psychopathology, other substance use) and examine the impact of such factors on social cognition outcomes. Second, the cross-sectional design of the current study prevented exploration of whether social cognitive alterations emerge or dissipate with continued cannabis use and/or changes in chronicity of use, and severity of CUD; a notion to be confirmed by longitudinal designs. Third, participants endorsed a range of CUD severities (i.e., mild, moderate, severe) and it may be that social cognitive deficits only emerge in severe dependence. This was tested via our secondary aim which found no significant association between CUDIT-R scores and social cognition thus there does not appear to be a significant association between CUD severity and social cognitive outcomes. However, more research is required to replicate these findings. Fourth, our study had an insufficient sample size to examine the moderating effects of other sample characteristics (e.g., schizotypal traits, SPQ and psychotic experiences, CAPE) shown to be associated with social cognition (Weinreb et al. 2022). It may be that other psychopathology variables moderate the association between cannabis use and social cognition. Future studies should confirm the role of which moderators affect the association between CUD and social cognition using larger samples. Fifth, although the current study used a semi-structured interview and validated assessment tool (i.e., TLFB) to measure cannabis use metrics, we did not utilise a standardised questionnaire to measure cannabis use nor did we test samples of the cannabis consumed by participants to determine Δ9-THC concentration. Future research should utilise validated semi-structured interviews such as the Daily Sessions, Frequency, Age of Onset, and Quantity of Cannabis Use Inventory (DFAQ-CU) alongside the TLFB and cannabis sample testing for Δ9-THC concentration to enhance methodological robustness and reproducibility.

### Conclusion

The current study found that social cognition (i.e., emotion recognition, emotion differentiation, immediate and delayed face memory), did not differ between CUD and controls, nor did level of problematic cannabis use (i.e., CUDIT-R) or cannabis dosage (i.e., cannabis grams/past month) significantly predict social cognition in the CUD group, accounting for confounds. These findings may indicate that altered social cognition is not a key feature of CUD, or that the relationship between CUD and social cognition is moderated by factors such as age, treatment seeking, education, IQ, and comorbidities (e.g., psychopathology, other substance use) which were unable to be examined in the current sample due to possible sampling bias. Indeed, the current CUD sample were young, non-treatment seeking, endorsed no major comorbidities, and had an above average IQ which may have served as a protective factor against social cognitive alterations; however, these results need to be confirmed by replication studies. Future research is required that examines social cognition in vulnerable CUD samples from the general community and treatment services who endorse a broader range of ages, educational experiences, IQ, and comorbidities. Such new research will provide insight into whether social cognition is a feature of CUD, or more vulnerable CUD populations, which will support the development of clinical interventions to mitigate potential interpersonal and social challenges.

## Supplementary Information

Below is the link to the electronic supplementary material.

Supplementary Material 1 (DOCX 51 KB)

## Data Availability

Not applicable.

## Glossary

AUDIT: Alcohol Use Disorder Identification Test.
BAC: Blood Alcohol Concentration.
CAPE: Community Assessment of Psychic Experiences.
CB: Cannabis.
CUD: Cannabis Use Disorder.
CUDIT-R: Cannabis Use Disorder Identification Test - Revised.
DHQ: Drug History Questionnaire.
DSM-5-TR: Diagnostic and Statistical Manual for Mental Disorders – 5th Edition – Text Revision.
ER: Emotion Recognition.
FM: Face Memory - immediate.
FM-d: Face Memory - delayed.
FTND: Fagerstrom Test of Nicotine Dependence.
hCG: Human Chorionic Gonadotropin.
IQ: Intelligence Quotient.
MED: Emotion Differentiation.
MDMA: 3,4-Methyl​enedioxy​methamphetamine.
MINI-5: Mini International Neuropsychiatric Interview – 5th Edition.
Penn CNB: Pennsylvania Computerised Neurocognitive Battery.
SCID-5-RV: Structured Clinical Interview for the Diagnostic and Statistical Manual of Mental Disorders – 5th Edition – Research Version.
SPQ: Schizotypal Personality Questionnaire.
STAI: State-Trait Anxiety Inventory.
Δ9-THC: delta-9-tetrahydrocannabinol.
TLFB: Timeline Follow Back.
WASI-II: Weschler Abbreviated Scale of Intelligence – 2nd Edition.
