# Supplementary material for: Social cognition in young adults who endorse a cannabis use disorder
Source: Psychopharmacology (Berl). 2025 Sep 17;243(4):861–71. doi: 10.1007/s00213-025-06890-z (PMC13035583; doi:10.1007/s00213-025-06890-z)
Supplement: Supplementary file 1 — Supplementary Material 1 (DOCX 51 KB) [file 213_2025_6890_MOESM1_ESM.docx]

**Social cognition in young adults who endorse a cannabis use disorder.**

Gabrielle Abbott^a^, Lisa Greenwood^b^, PhD, Jessica G. Bartschi^a^, PhD, Suraya Dunsford^a,c,d^, Isabella Goodwin^e^, Anastasia Paloubis^e^, Marianna Quinones Valera^e^, M.A, Eugene McTavish^e^, PhD, Antonio Verdejo-Garcia^f^, PhD, Janna Cousijn^g^, PhD, Gary C. K. Chan^h^, PhD, Nadia Solowij^a^, PhD, Valentina Lorenzetti^e^, MSc, PhD

^a^ School of Psychology, Faculty of the Arts, Social Sciences, and Humanities, University of Wollongong, Wollongong, Australia
^b^ School of Medicine and Psychology, The Australian National University, Canberra, Australia
^c^ School of Psychology, Faculty of Health, University of Plymouth, Plymouth, UK.

^d^ Brain Research and Imaging Centre, Faculty of Health, University of Plymouth, Plymouth, UK.
^e^ Neuroscience of Addiction and Mental Health Program, Healthy Brain and Mind Research Centre, School of Health and Behavioural Sciences, Faculty of Health Sciences, Australian Catholic University, Fitzroy, Australia.
^f^ School of Psychological Sciences and Turner Institute for Brain and Mental Health, Monash University, Melbourne, Australia
^g^ Neuroscience of Addiction Lab, Department of Psychology, Education & Child Studies, Erasmus University Rotterdam, The Netherlands
^h^ National Centre for Youth Substance Use Research, University of Queensland

**Corresponding author:**

Professor Valentina Lorenzetti, PhD
Neuroscience of Addiction and Mental Health Program, Healthy Brain and Mind Research Centre, School of Behavioural and Health Sciences, Faculty of Health Sciences, Australian Catholic University
Address: Level 5 Daniel Mannix Building, 115 Victoria Parade, Fitzroy VIC 3065, Australia
Email: [valentina.lorenzetti@gmail.com](mailto:valentina.lorenzetti@gmail.com)
Phone number: +61 03 9230 808

**Supplementary Materials**

1. **Supplementary methods**

1.1 Online screening

Interested participants were asked to complete an online questionnaire to ensure basic demographic (e.g., age) and cannabis use parameters (regular cannabis users: frequency of cannabis use in the past 12-months; controls: recency and lifetime cumulative cannabis use) met study inclusion criteria. The FTND (Fagerström, 2012) and AUDIT (Babor et al., 2001) were used to screen for self-reported severe nicotine and alcohol dependence, respectively. The online screening questionnaire took approximately 10-minutes to complete and after all questions were answered, participants were invited to leave their contact details to be contacted by a member of the research team to complete further screening via telephone.

1.2 Telephone screening

The telephone screen was used to confirm self-reported online screening measures. The MINI-V (Sheehan et al., 1998) was administered at the telephone screening stage to identify participants meeting exclusionary criteria for severe alcohol use disorder and additional exclusionary psychiatric diagnoses. The SCID-V-RV (First et al., 2015) was administered to cannabis using individuals to screen eligibility for CUD criteria (≥ 2 criteria). Additional information about the study was provided, and participants were given the opportunity to ask questions prior to study invitation acceptance. Individuals who verbally consented to participate in the study were sent a confirmation email with an information pack attached which included a copy of the participant information sheet, consent forms, directions to testing facilities, and appointment details.

1.3 Inclusion/exclusion criteria assessment

As reported in the main text, some participants endorsed elevated levels of recreational substance use (i.e., other than cannabis for the CUD group) but were retained for sample generalisability and representativeness. The details of the other substance use violation and associated demographic information is provided in Supplementary Table 1.

**Supplementary Table 1**. Other substance use above recreational levels.

| **Substance** | **Group** | **Age** | **Sex** | **Substance use violations** | | |
| --- | --- | --- | --- | --- | --- | --- |
|  |  | *(years)* |  | *^1^Regular use* | *^2^Recency of use (days)* | *^3^Cumulative lifetime exposures* |
| *Ecstasy* |  |  |  |  |  |  |
|  | Mild | 20.65 | Female | - | 18 days | - |
|  | Mild | 21.64 | Male | - | 30-days  (+ve in urine) | - |
|  | Mild | 24.09 | Male | - | - | 50 |
|  | Mild | 26.39 | Male | - | 22 days | - |
|  | Moderate | 19.93 | Male | - | 21 days | - |
|  | Moderate | 20.35 | Male | - | - | 150 |
|  | Moderate | 24.91 | Male | - | 17 days | 100 |
|  | Moderate | 29.69 | Male | - | - | 80 |
|  | Moderate | 24.63 | Female | - | - | 50 |
|  | Severe | 26.22 | Male | - | 14 days | - |
|  | Severe | 20.85 | Male | - | 14 days | - |
|  | Severe | 28.44 | Male | - | - | 50 |
|  | Severe | 22.17 | Male | - | 12 days | - |
|  | Severe | 26.74 | Male | - | 21 days |  |
|  | Severe | 25.07 | Male | - | 18 days |  |
|  | Severe | 21.87 | Female | - | 10 days |  |
|  | Severe | 28.30 | Male | - | - | 70 |
| *Amphetamine* |  |  |  |  |  |  |
|  | Severe | 22.88 | Male | Yes | 1 day | >90 |
| *Nitrous Oxide* |  |  |  |  |  |  |
|  | Mild | 21.64 | Male | - | 14 days | - |
|  | Mild | 29.85 | Male | - | - | 50 |
|  | Moderate | 19.93 | Male | - | - | 57 |
|  | Moderate | 20.35 | Male | - | - | 400 |
|  | Moderate | 21.10 | Male | - | 2 days | - |
|  | Severe | 22.17 | Male | - | 12 days | *-* |
|  | Severe | 26.74 | Male | - | 14 days | - |
|  | Severe | 25.07 | Male | - | - | >1000 |
|  | Severe | 21.87 | Female | - | 5 days | 50 |
| *Cocaine* |  |  |  |  |  |  |
|  | Mild | 26.39 | Male | - | 24 days | - |
|  | Mild | 26.88 | Female | - | 21 days  (+ve in urine) | - |
|  | Severe | 20.85 | Male | - | 21 days | - |
|  | Severe | 22.64 | Male | - | - | 50 |
|  | Severe | 21.87 | Female | - | 4 days | - |
|  | Severe | 19.99 | Male | - | 18 days | - |
|  | Severe | 28.30 | Male | - | 21 days | - |
| *Hallucinogen* |  |  |  |  |  |  |
|  | Mild | 20.83 | Male | - | 3 days | - |
|  | Mild | 24.71 | Male | - | 25 days | - |
|  | Moderate | 19.93 | Male | - | 14 days | - |
|  | Moderate | 23.88 | Male | - | 24 days | - |
|  | Moderate | 27.72 | Female | - | 21 days | - |
|  | Severe | 22.17 | Male | - | 12 days | - |
|  | Severe | 22.05 | Male | - | 5 days | - |
|  | Severe | 22.88 | Male | - | 2 days | - |
|  | Severe | 20.07 | Male | - | - | 150 |
| *Methamphetamine* |  |  |  |  |  |  |
|  | Mild | 26.39 | Male | - | 3 days | - |
|  | Severe | 26.22 | Male | - | - | 10^4^ |
| *Ketamine* |  |  |  |  |  |  |
|  | Severe | 20.85 | Male | - | 14 days | - |
|  | Severe | 25.07 | Male | - | 17 days | - |
| *Benzodiazepine* |  |  |  |  |  |  |
|  | Severe | 22.64 | Male | - | *missing*  (+ve in urine) | - |

**Note:** group (control, mild, moderate, severe); ^1^weekly use for >3-months for any illicit substance (other than cannabis) within the past 5-years; ^2^use of any illicit substance other than cannabis within 30-days prior to testing; ^3^cumulative lifetime episodes >50; ^4^cumulative lifetime use >5 for methamphetamine or any drug administered intravenously.

1.4 Normality and statistical assumption checks

Prior to examining group differences for any variables, assumptions of normality, linearity, homogeneity of variance, and homogeneity of regression slopes were inspected using visual plots and relevant statistics (sections 1.4.4-1.4.5).

*1.4.1 Covariates*

Several covariates violated statistical assumptions (i.e., AUDIT, STAI-Trait, FTND, hours from last cannabis use), and were handled as follows. First, the covariates AUDIT and STAI-Trait violated the assumption of homogeneity of regression slopes; therefore, group comparisons were run with and without all covariates to evaluate their impact on the results (i.e., AUDIT, STAI-Trait, FTND). Second, the distribution of FTND scores and hours since last cannabis use were non-normal and skewed. Consequently, these two variables were recoded using a median split approach (i.e., hours from cannabis last use being ≤ 15 hours and ≥ 15.25 hours; and FTND scores being < 1 and ≥ 1).

*1.4.2 Outcome variables*

The distribution of delayed face memory (i.e., FM-d) was skewed, and statistics violated the assumption of normality even after the removal of outliers. Therefore, robust regressions were run for this outcome variable. The outcome variables for emotion recognition (i.e., ER) and immediate face memory (i.e., FM) showed mild violations of normality based on Shapiro-Wilk statistics. However, their distributions were approximately normal, and their skewness and kurtosis were within the normal range of -2 to +2, and therefore, parametric tests were deemed appropriate. The outcome variable for emotion differentiation (i.e., MED) was normally distributed.

*1.4.3 Regression predictor variables*

Normality was violated for cannabis grams/past month, which was significantly skewed and non-normal. Cannabis grams/past month was therefore recoded into a categorical variable with four levels which gave clinically meaningful consumption levels that discriminate varying levels of exposure (i.e., < 7 gm/past month, 7 - 11.5 gm/past month, 11.75 – 28 gm/past month, > 28 gm/past month).

*1.4.4 Group analyses: ANCOVA*

1. *Normality*: Inspection of histograms and Shapiro-Wilk statistics showed that data was normally and approximately normally distributed. ANCOVA is robust to moderate violations or normality, thus the statistical approach was retained.
2. *Linearity:* Inspection of scatterplots indicated that the relationship between covariates (AUDIT, FTND, STAI-Trait) and the dependent variable (socioemotional cognition efficiency score: ER, MED, FM) was linear.
3. *Homogeneity of regression slopes*: The assumption for homogeneity of regression slopes was upheld for FTND and the independent variable (i.e., Group: CUD, control). However, there were significant interactions between AUDIT and Group, and STAI-Trait and Group.
4. *Homogeneity of variance:* Levene’s tests were non-significant.

*1.4.5 Correlations: Multiple Linear Regression*

1. *Normality:* Not all continuous variables were approximately normally distributed based on inspection of histograms and Shapiro-Wilk statistics. Of the predictors, CUDIT was normally distributed but dosage (i.e., cannabis grams/past month) was not – this variable was recoded into a categorical variable with four levels that was carefully selected to retain natural variance. Of the covariates, STAI-Trait and AUDIT were normally and approximately normally distributed with no extreme outliers, respectively. However, FTND and recency of cannabis use (i.e., hours since last cannabis use) were not normally distributed and required recoding; a median split approach was used to recode these variables into categorical variables with two levels.
2. *Outliers:* No extreme multivariate outliers were detected as per comparison of the Maximum Mahalanobis Distance statistics for each outcome variable the critical chi-square value (all values < χ^2^ = 22.458).
3. *Multicollinearity:* Inspection of tolerance (i.e., >.01) and VIF (i.e., < 5) indicated that multicollinearity would not interfere with interpreting the outcome of the regressions.
   1. *Tolerance: predictors with tolerances < .01 are multicollinear with one or more other predictors and is cause for concern; all Tolerance statistics were > .600.*
   2. *VIF: predictors with VIFs > 5 are multicollinear with one or more other predictors; all VIF statistics were < 2.*
4. *Normality, Linearity, and Homoscedasticity of residuals:* Inspection of the normal probability plot of standardised residuals as well as the scatterplot of standardised residuals against standardised predicted values indicated that the assumptions of normality, linearity, and homoscedasticity of residuals were met as there were no clear patterns in the spread of data points.
5. **Supplementary results**

**Supplementary Table 2.** Outliers removed from sensitivity analysis.

|  | **Efficiency score** | **Group** | **Age** | **Sex** |
| --- | --- | --- | --- | --- |
| **ER** |  |  |  |  |
|  | LD043 | Control | 27.06 | Male |
|  | LD016 | Mild | 29.85 | Male |
|  | LD031 | Moderate | 23.74 | Male |
|  | LD023 | Severe | 21.68 | Male |
|  | LD085 | Severe | 22.64 | Male |
| **MED** |  |  |  |  |
|  | LD031 | Moderate | 23.74 | Male |
|  | LD061 | Severe | 21.67 | Male |
| **FM** |  |  |  |  |
|  | LD080 | Control | 20.31 | Male |
|  | LD112 | Moderate | 27.72 | Female |
|  | LD115 | Moderate | 22.24 | Male |
|  | LD085 | Severe | 22.64 | Male |
| **FM-d** |  |  |  |  |
|  | LD080 | Control | 20.31 | Male |

- 1. Group differences in socioemotional - sensitivity analyses.

*2.1.1 Outlier removal*

Sensitivity analyses removing outliers resulted in no significant changes to models: emotion recognition (*F* (1,95) = 2.12e-4, *p* = .988, *d=*0; emotion differentiation (*F* (1,98) = 1.713, *p* = .194, *d=*.263); and face memory - immediate (*F* (1,98) = .471, *p* = .494, *d=.*142).

**Supplementary Table 3.** Sensitivity analysis - socioemotional cognition differences between CUD and controls after removal of covariates.

|  |  | **CUD** *M (SD)* | **Control**  *M (SD)* | **Group comparisons**  t/χ^2^/*F, p, d* |
| --- | --- | --- | --- | --- |
| Emotion recognition, ER |  | 0.019 (1.52) | -0.049 (1.48) | 0.047^111^, .829, 0 |
| Emotion differentiation, MED |  | -0.198 (1.34) | 0.508 (1.27) | 6.59^112^, .**012**, 0.487 |
| Face memory |  |  |  |  |
| immediate, FM |  | -0.037 (1.47) | 0.095 (1.62) | 0.175^113^, .677, 0.090 |
| delayed, FM-d |  | -0.065 (1.26) | 0.168 (1.37) | -0.819^104^, .415, - |

**Note:** bold = *p* < .05; - = NA or not provided by statistical output; *d =* Cohen’s *d*^*^Cohen (1988) reports the following intervals: 0 - .2: very small effect; .2 to .5: small effect; .5 to .8: medium effect; .8 and higher: large effect.

- 1. CUDIT scores and cannabis dosage as predictors of socioemotional cognition – sensitivity analyses.

*2.2.1 Outlier removal*

Sensitivity regression analyses removing outliers resulted in no significant changes to models: emotion recognition (*F* (6,52) = 1.236, *p* = .303), emotion differentiation (*F* (6,53) = 0.439, *p* = .849); and face memory - immediate (*F* (6,55) = 0.615, *p* = .717).

**Supplementary Table 4.** CUDIT-R and cannabis dosage as predictors of socioemotional cognition in CUD.

|  |  | **Model** | **Predictors** |  | **Covariates** |  |  |  |
| --- | --- | --- | --- | --- | --- | --- | --- | --- |
|  |  |  | CUDIT | CB grams/past month | AUDIT | STAI-Tait | FTND | last CU hours |
|  |  | adjusted *R^2^, F^df^/t^df^, p* | *t^df^, p* | *t^df^, p* | *t^df^, p* | *t^df^, p* | *t^df^, p* | *t^df^, p* |
| Emotion recognition | ER | .047, 1.492^54^, .198 | 0.515^54^, .608 | 0.415^55^, .680 | 1.368^54^, .177 | -1.693^54^, .096 | 1.758^54^, .084 | 0.323^54^, .748 |
| Emotion differentiation | MED | -.042, 0.594^55^, .734 | -0.307^55^, .760 | 0.116^55^, .908 | 0.367^55^, .715 | -1.339^55^, .186 | 0.695^55^, .490 | 0.845^55^, .402 |
| Face memory, immediate | FM | -.022, 0.779^55^, .590 | 0.071^55^, .944 | 1.573^55^, .122 | -0.383^55^, .703 | -0.702^55^, .486 | .977^55^, .333 | 0.925^55^, .359 |
| delayed | FM-d | - | -0.834^55^, .408 | 1.368^55^, .177 | -0.030^55^, .976 | -1.302^55^, .198 | 0.840^55^, .404 | 0.815^55^, .419 |

**Note:** bold = *p* < .05; - = not provided by statistical output; CB = cannabis; CU = cannabis use.

**Supplementary Table 5.** Sensitivity analysis – CUDIT-R and cannabis dosage as predictors of socioemotional cognition in CUD after removal of covariates.

|  |  | **Model** | **Predictors** |  |
| --- | --- | --- | --- | --- |
|  |  |  | CUDIT | CB grams/past month |
|  |  | adjusted *R^2^, F^df^/t^df^, p* | *t^df^, p* | *t^df^, p* |
| Emotion recognition | ER | -.026, 0.233^58^, .793 | 0.506^58^, .614 | -.124^58^, .902 |
| Emotion differentiation | MED | -.024, 0.274^59^, .761 | -0.428^59^, .670 | -0.289^59^, .774 |
| Face memory, immediate | FM | .009, 1.281^60^, .285 | 0.024^60^, .981 | 1.349^60^, .182 |
| delayed | FM-d | - | -0.874^59^, .386 | 1.45^59^, .152 |

**Note:** bold = *p* < .05; - = not provided by statistical output; CB = cannabis.

**References**

Babor, T. F., Higgins-Biddle, J. C., Saunders, J. B., & Monteiro, M. G. (2001). *AUDIT: The Alcohol Use Disorders Identification Test: guidelines for use in primary health care*. World Health Organization.

Fagerström, K. (2012). Determinants of tobacco use and renaming the FTND to the Fagerstrom Test for Cigarette Dependence. *Nicotine & Tobacco Research: Official Journal Of The Society For Research On Nicotine And Tobacco, 14*(1), 75-78. <https://doi.org/10.1093/ntr/ntr137>

First, M. B., Williams, J. B. W., Karg, R. S., & Spitzer, R. L. (2015). Structured Clinical Interview for DSM-5 - Research Version (SCID-5 for DSM-5, Research Version); SCID-5-RV, Version 1.0.0).

Sheehan, D. V., Lecrubier, Y., Sheehan, K. H., Amorim, P., Janavs, J., Weiller, E., Hergueta, T., Baker, R., & Dunbar, G. C. (1998). The Mini-International Neuropsychiatric Interview (M.I.N.I.): the development and validation of a structured diagnostic psychiatric interview for DSM-IV and ICD-10. *The Journal of Clinical Psychiatry, 59*, 22-33.
